# Effects of *Rhizophagus intraradices* on soybean yield and the composition of microbial communities in the rhizosphere soil of continuous cropping soybean

**DOI:** 10.1038/s41598-022-22473-w

**Published:** 2022-10-17

**Authors:** Weiguang Jie, Dongying Yang, Yanxuan Yao, Na Guo

**Affiliations:** 1 Engineering Research Center of Agricultural Microbiology Technology, Ministry of Education, Heilongjiang University, Harbin, 150500 China; 2 Key Laboratory of Microbiology, College of Life Sciences, Heilongjiang University, Harbin, 150080 China; 3 School of Food Engineering, Heilongjiang East University, Harbin, 150066 China

**Keywords:** Biodiversity, Agroecology

## Abstract

Soybean (*Glycine max* L.) is an important oil and economic crop in the world. However, soybean continuous cropping may lead to the decline of soybean yield and quality. The purpose of this study was to investigate the effects of *Rhizophagus intraradices* on soybean growth/yield, root rot disease index, and the composition of microbial communities in the rhizosphere soil of continuous cropping soybean at the R8 stage. The results showed that the 100-seed weight, seed-yield per plant, yield per 0.04 hectare, pods per plant, seed number per plant, branch number, plant height, and fresh weight of root and shoot, and disease index of soybean root rot were significantly affected by the inoculation of *R. intraradices* and soybean continuous cropping. The growth/yield indexes of soybean were the highest in the inoculated soybean plants under non-continuous cropping. Inoculation of *R. intraradices* and soybean continuous cropping significantly decreased and increased the disease index of soybean root rot, respectively. Bacterial diversity levels in the rhizosphere soil of continuous cropping soybean were lower than those in non-continuous cropping soybean. Furthermore, it also showed that inoculation of *R. intraradices* could increase the bacterial and fungal diversity in rhizosphere soil of soybean. It also showed that both inoculation and soybean continuous cropping had effects on the composition of microbial communities in the rhizosphere soil of soybean. Proteobacteria and Ascomycota were the most dominant bacterial and fungal phylum in all samples, respectively. The results would contribute to evaluating the biocontrol potential of *R. intraradices* against soybean root rot disease, increase soybean yield and improve the composition of microbial communities in the rhizosphere soil of continuous cropping soybean.

## Introduction

Soybean (*Glycine max* L.) is an important oil crop in China, providing abundant lipid and protein resources for the human diet^1,2^. Heilongjiang is the main soybean production area in China, and the soybean planting area and yield both play an important role in China’s soybean industry^3^. However, continuous cropping in soybean is increasingly practiced in Heilongjiang Province, leading to substantial yield reductions and quality degradation. The obstacles of soybean continuous cropping have become the major limiting factors for the high and stable yield of soybean^4,5^. Soybean root rot is a common destructive disease in a continuous cropping system. Soybean root rot is one of the major limiting factors of soybean continuous cropping in Northeast China. The main pathogens of soybean root rot in Heilongjiang Province are *Fusarium*, *Rhizoctonia*, *Phytophthora*, *Mortierella*, and *Pythium*, among which *Fusarium oxysporum* is the main pathogenic fungus of soybean root rot^6^.

Continuous cropping system resulted in the changes of the composition of microbial communities in rhizosphere soil, enrichment of secondary metabolites in soil, and soil enzyme activities, etc.^5,7^. Arbuscular mycorrhizal (AM) fungi are oligotrophic microbes that live with the roots of more than 90% of terrestrial plants including the vast majority of crops^8,9^. They can significantly improve the absorption of nutrient elements by host plants and enhance the activities of defense-related enzymes, thus promoting plant growth and enhancing disease resistance and stress resistance of plants^10,11^. They also play an important role in maintaining ecosystem diversity and microecosystem stability^12,13^. They have been widely used in different crops and have been shown to improve plant growth^14–17^. They synergistically interact with *Trichoderma*, *Gliocladium*, *Streptomyces*, and *Pseudomonas* to reduce the incidence of soil-borne diseases^18^. In the rhizosphere ecosystem, AM fungi and growth-promoting rhizobacteria play an important role in regulating the incidence of soil-borne diseases and the availability of nutrients. *F. oxysporum* was significantly decreased in soybean plants inoculated with *Funneliformis mosseae*^19^. In addition, our previous research has demonstrated that *Fu. mosseae* can affect plant growth, disease index of soybean root rot and the composition of microbial communities in the roots and rhizosphere soil in a continuous cropping system^20,21^. However, the effects of *R. intraradices* on the composition of microbial communities in the rhizosphere soil under continuous cropping system at reproductive R8 stage have never been reported.

The purposes of this study were to investigate the effects of inoculation of *R. intraradices* on soybean growth/yield, root rot disease index, as well as on the composition of microbial communities in the rhizosphere soil under continuous cropping and non-continuous cropping system at the R8 stage. The following hypotheses were tested in this study: (1) soybean growth/yield and root rot disease index would increase and decrease after inoculation of *R. intraradices*, respectively; (2) soybean growth/yield and root rot disease index would decrease and increase under soybean continuous cropping, respectively; and (3) inoculation of *R. intraradices* could impact the composition of microbial communities in the rhizosphere soil compared with non-inoculated rhizosphere soil. This study was planned to evaluate the biocontrol potential of *R. intraradices* against soybean root rot disease as well as its role in alleviating the obstacles of soybean continuous cropping.

## Materials and methods

### Soybean cultivar and AM fungi inoculum

Soybean (*Glycine max* L.) cv. Heinong 48 (19.50% average fat content and 45.23% average protein) was used in this study. The soybean cultivar was purchased from Heilongjiang Academy of Agricultural Sciences, Harbin, China. The soybean cultivar has been widely planted in the Heilongjiang Province of China.

The AM fungus (*Rhizophagus intraradices*) was originally obtained from the rhizosphere soil of continuous cropping system in Heilongjiang Province of China by our research group^22^. The biological characteristics of AM fungus (*R. intraradices*) were similar to those of Yang et al.^23^. *R. intraradices* develops high extraradical mycelium length and spore production. The AM fungus was propagated in pot culture with alfalfa plants grown in sterilized vermiculite, river sand and soil (3:2:5, v/v/v) for about 5 months^22^. The AM colonization (92.5%) and spore density (510 per 10 g of air-dried soil) were determined after harvest. The AM colonization was estimated according to Phillips and Hayman^24^. The general inoculum consisted of *R. intraradices* colonized root fragments, hyphae, spores, and substrate.

### Experimental design

The experimental site was situated in the experimental fields of Heilongjiang East University, Heilongjiang Province, China (126° 36′ E, 45° 39′ N). Annual precipitation is about 569.1 mm with almost 81% occurring in May to September, and annual average temperature is about 20.6 °C during growing season. The field experiment was designed as a randomized block design, three times replicated with AM fungal treatments (non-inoculated and inoculated with *R. intraradices*) and continuous cropping regimes (0 and 1 year of continuous cropping for soybean) as factors. 0 year of continuous cropping for soybean means that the field has never been planted with soybean, but only corn. 1 year of continuous cropping for soybean means that the field was planted with soybean only last year, and soybean will continue to be planted this year. The field soils have not been fertilized in recent 2 years. Non-organic soybean fields are usually applied with (NH_4_)_2_HPO_4_ 150–180 kg, KCl 60–80 kg and CO(NH_2_)_2_ 30–50 kg per hectare in Heilongjiang Province, China. The physicochemical properties of the field soils were given in Table 1. Twelve 20 m × 20 m plots (four treatments × three replicates) close to each other with 2 m of buffer zones between each plot were established. A spacing of 50 cm between rows and soybean plants was maintained.

**Table 1:** Soil physicochemical properties.

Continuous cropping regimes	Organic matter (g/kg)	Total nitrogen (g/kg)	Total phosphorus (g/kg)	Total potassium (g/kg)	Ammonium nitrogen (mg/kg)	Nitrate nitrogen (mg/kg)	pH
0Y	25.58 ± 0.28	2.59 ± 0.17	5.64 ± 0.19	31.77 ± 0.30	1.17 ± 0.06	0.93 ± 0.06	7.47 ± 0.02
1Y	28.27 ± 0.32	2.47 ± 0.16	5.41 ± 0.13	33.48 ± 0.31	1.06 ± 0.05	0.82 ± 0.04	7.19 ± 0.03

0Y and 1Y represent 0 year and 1 year of continuous cropping, respectively.

### Field experiment and sample collection

Soybean seeds were first surface-sterilized with 70% (v/v) ethanol for 3 min, subsequently disinfected with 3% (v/v) sodium hypochlorite for 3 min, finally washed with sterilized distilled water and air dried. Soybean seeds were sown on May 18, 2019. Before sowing the soybean seeds in the field, 5 g of AM fungi inoculum was added to the corresponding field at a soil depth of 2–3 cm below each three soybean seeds^25,26^. The spore density was approximately 2295 per square meter. Three soybean seeds were placed into one location with a spacing of 50 cm apart from the other location. After 5–6 days they were thinned to one seedling per location. The plant population in plants was 40,000 per hectare. Soybean plants were regularly irrigated with tap water during the whole growth period. Day to flowering (R1, day from emergence to first open flower in 50% of the plants) on July 17, 2019. Day to maturity (R8, 95% of the pods have turned their mature color in 50% of the plants) on September 26, 2019.

The seeds, pods, shoots, roots and rhizosphere soils of twenty soybean plants were randomly collected for each treatment at the R8 stage on September 26, 2019. The maturity was assessed according to Fehr and Caviness^27^. The roots were repeatedly washed with running water to remove adhering soil, and then used to determine the disease index of soybean root rot. For the collection of soybean rhizosphere soils, the rhizosphere soils attached to soybean roots were collected with a brush and mixed thoroughly and stored at − 80 °C for analysis of the composition of microbial communities.

### Analysis of soybean growth/yield and disease index

The effects of *R. intraradices* on soybean plant growth was assessed by 100-seed weight, seed-yield per plant, yield per 0.04 hectare, pods per plant, seed number per plant, seed number per pod, node number, branch number, plant height, and fresh weight of root and shoot. The disease index of soybean root rot was determined according to Zhou et al.^28^. Plants were scored for disease as follows: 0. no disease spots on the basal stem and axial root; 1. sporadic disease spots present; 2. flakey sporadic disease spots present; 3. diseased areas present on 25% of the root length; 4. diseased areas present on 33% of the root length and disease spots coalesce around the stem, but root not necrotic; and 5. diseased areas present on > 50% of the root length.

### Soil genomic DNA extraction and high-throughput sequencing

Soil genomic DNA was extracted from 0.25 g of each rhizosphere soil sample using a PowerSoil DNA Isolation Kit (MOBIO Laboratories Inc., Carlsbad, CA, USA) according to the manufacturer’s instructions. The soil genomic DNA was purified with a PowerClean DNA Clean-up Kit (MOBIO Laboratories Inc., Carlsbad, CA, USA) and checked on 1.0% (w/v) agarose gel. The concentration and quality of the extracted genomic DNA were checked using a NanoDrop 2000 Spectrophotometer (Thermo Scientific, USA). The extracted genomic DNA was stored at − 20 °C. The variable V3–V4 regions of bacterial 16S rDNA were amplified from genomic DNA using the universal bacterial primers 335F (5ʹ-CADACTCCTACGGGAGGC-3ʹ) and 769R (5ʹ-ATCCTGTTTGMTMCCCVCRC-3ʹ)^29^. Fungal internal transcribed spacer 1 (ITS1) region was amplified from genomic DNA using the universal fungal primers ITS1F (5ʹ-CTTGGTCATTTAGAGGAAGTAA-3ʹ) and ITS2 (5ʹ-GCTGCGTTCTTCATCGATGC-3ʹ)^30^. The PCR mixture consisted of each 5 µM primer 0.8 μL, 2.5 mM dNTP 2.0 μL, FastPfu Buffer 4.0 μL, FastPfu Polymerase 0.4 μL, template DNA 10 ng, and ddH_2_O in a total volume of 20 μL. The PCR cycling conditions were as follows: 95 °C for 5 min, followed by 30 cycles of 95 °C for 30 s, 50 °C for 30 s and 72 °C for 40 s, and a final extension at 72 °C for 7 min. The PCR products were purified using a GeneJET Gel Extraction Kit (Thermo Scientific, Waltham, Massachusetts, USA), quantified using QuantiFluor™-ST (Promega, Madison, WI, USA), and then pooled at equal concentrations. Amplicon sequencing was performed on the Illumina HiSeq 2500 platform (BioMarker Technologies Co., Ltd., Beijing, China). All raw reads have been deposited into the National Center for Biotechnology Information (NCBI) under the BioProject accession number PRJNA862608 and PRJNA862612.

### Sequence analysis

The raw sequences were processed using QIIME v1.8.0^31^. The paired-end sequences were joined with FLASH v1.2.7^32^. The low-quality sequences < 200 bp and with an average base quality score < 20, or containing ambiguous bases were removed before further analysis. Chimeras were detected and eliminated using UCHIME^33^. High-quality sequences with similarities ≥ 97% were clustered into one operational taxonomic unit (OTU)^34^. The OTUs were classified and identified by the BLAST algorithm-based search within GenBank (http://blast.ncbi.nlm.nih.gov/Blast.cgi). The Ace (http://www.mothur.org/wiki/Ace), Chao1 (http://www.mothur.org/wiki/Chao), Shannon (http://www.mothur.org/wiki/Shannon), Simpson (http://www.mothur.org/wiki/Simpson), and Good’s Coverage (http://www.mothur.org/wiki/Coverage) were used to evaluate the microbial community richness and diversity and were measured using MOTHUR v.1.30. The distributions of common and unique OTUs based on 97% sequence similarities were shown in VENN diagrams^35^. Beta diversity analysis was performed according to the Bray–Curtis distance calculation method, and Principal coordinates analysis (PCoA) was used to analyze the composition of bacterial and fungal communities in the rhizosphere soil of continuous cropping soybean. The composition of microbial communities also was analyzed and compared among the four rhizosphere soil samples so that histograms could be drawn of the composition of microbial communities of multiple samples at the phylum level and the genus level, respectively. Two heatmaps were drawn to display the relative differences in OTU abundances among the four rhizosphere soil samples using the pheatmap package v1.0.2 in R/Bioconductor (https://cran.r-project.org/web/packages/pheatmap/index).

### Statistical analysis

Analysis of variance (ANOVA) and Duncan’s tests (honestly significant differences, HSD) was applied to evaluate significant differences between treatments (*P* < 0.05) using SPSS 20.0 (SPSS Inc., Chicago, Illinois, USA). Factorial design was used to analyze the interactive effects of the inoculation of *R. intraradices* and continuous cropping regimes.

### Legal permission

Field studies on our plants, including the collection of plant material, comply with relevant institutional, national, and international guidelines and legislation.

## Results

### Effects of *R. intraradices* and soybean continuous cropping on soybean growth/yield

The 100-seed weight, seed-yield per plant, yield per 0.04 hectare, pods per plant, seed number per plant, seed number per pod, node number, branch number, plant height, and fresh weight of root and shoot were decreased by soybean continuous cropping (Table 2). In addition, the 100-seed weight, seed-yield per plant, yield per 0.04 hectare, pods per plant, seed number per plant, branch number, plant height, and fresh weight of root and shoot were significantly increased by the inoculation of *R. intraradices*. As shown in Table 2, the growth/yield indexes of soybean were the highest in the inoculated soybean plants under non-continuous cropping.

**Table 2:** Factorial design results of soybean growth/yield and disease index of soybean root rot.

Treatment number	1	2	3	4
Continuous cropping regimes	0Y	0Y	1Y	1Y
AM fungal treatments	Non	In	Non	In
100-seed weight (g)	24.27 ± 0.28^b^	27.46 ± 0.21^a^	18.62 ± 0.19^d^	21.91 ± 0.18^c^
Seed-yield per plant (g)	21.34 ± 0.87^b^	24.75 ± 1.03^a^	16.58 ± 0.94^d^	18.53 ± 1.05^c^
Yield per 0.04 hectare (kg)	34.23 ± 1.01^b^	39.57 ± 0.68^a^	26.65 ± 0.92^d^	29.44 ± 0.79^c^
Pods per plant	64.76 ± 1.94^b^	75.82 ± 1.68^a^	49.22 ± 2.02^d^	57.39 ± 1.40^c^
Seed number per plant	133.67 ± 5.03^b^	157.33 ± 6.03^a^	97.67 ± 6.50^d^	119.33 ± 4.73^c^
Seed number per pod	2.33 ± 0.58^a^	3.33 ± 0.58^a^	1.67 ± 0.58^b^	2.23 ± 0.68^b^
Node number	12.27 ± 0.56^a^	13.43 ± 0.80^a^	10.82 ± 0.92^b^	11.41 ± 0.58^b^
Branch number	8.31 ± 0.69^b^	9.87 ± 0.25^a^	6.44 ± 0.45^d^	7.79 ± 0.47^c^
Plant height (cm)	82.33 ± 1.52^b^	89.21 ± 1.08^a^	74.17 ± 0.97^d^	84.51 ± 1.19^c^
Root fresh weight (g)	11.21 ± 0.87^b^	16.52 ± 0.92^a^	8.46 ± 0.69^c^	11.42 ± 0.93^b^
Shoot fresh weight (g)	101.02 ± 1.91^b^	114.63 ± 1.57^a^	80.87 ± 1.32^c^	99.63 ± 1.86^b^
Root rot disease index	0.79 ± 0.03^b^	0.57 ± 0.03^c^	1.20 ± 0.03^a^	0.80 ± 0.03^b^

0Y and 1Y represent 0 year and 1 year of continuous cropping, respectively.

Non represents non-inoculated with *R. intraradices*. In represents inoculated with *R. intraradices*.

Different letters indicate significant differences from different treatments (*P* < 0.05).

The variance analysis of the factorial design showed that the growth/yield indexes of soybean were significantly affected by the inoculation of *R. intraradices* and continuous cropping regimes (*P* < 0.01) except seed number per pod and node number which were only affected by continuous cropping regimes (Table 3). It also showed that the interactive effects of the inoculation of *R. intraradices* and continuous cropping regimes were significantly affected the yield per 0.04 hectare, plant heigh, and fresh weight of root and shoot (*P* < 0.05).

**Table 3:** Variance analysis of the factorial design of soybean growth/yield and disease index of soybean root rot.

Index	Source	Sum of squares	df	Mean square	F-value	*P*-value	Significance level
100-seed weight (g)	Y	97.242	1	97.242	3076.471	0.000	**
	I	29.705	1	29.705	939.769	0.000	**
	Y × I	0.062	1	0.062	1.950	0.200	
	Error	0.253	8	0.032			
	Total	6537.051	12				
Seed-yield per plant (g)	Y	90.365	1	90.365	95.069	0.000	**
	I	21.520	1	21.520	22.641	0.001	**
	Y × I	1.577	1	1.577	1.659	0.234	
	Error	7.604	8	0.951			
	Total	5066.553	12				
Yield per 0.04 hectare (kg)	Y	235.056	1	235.056	317.815	0.000	**
	I	49.654	1	49.654	67.136	0.000	**
	Y × I	4.826	1	4.826	6.525	0.034	*
	Error	5.917	8	0.740			
	Total	12,949.012	12				
Pods per plant	Y	865.471	1	865.471	274.178	0.000	**
	I	277.345	1	277.345	87.862	0.000	**
	Y × I	6.264	1	6.264	1.984	0.197	
	Error	25.253	8	3.157			
	Total	47,001.504	12				
Seed number per plant	Y	4107.000	1	4107.000	130.037	0.000	**
	I	1541.333	1	1541.333	48.802	0.000	**
	Y × I	3.000	1	3.000	0.095	0.766	
	Error	252.667	8	31.583			
	Total	199,452.000	12				
Seed number per pod	Y	2.341	1	2.341	6.399	0.035	*
	I	1.841	1	1.841	5.032	0.055	
	Y × I	0.141	1	0.141	0.385	0.552	
	Error	2.927	8	0.366			
	Total	75.890	12				
Node number	Y	8.979	1	8.979	16.820	0.003	**
	I	2.306	1	2.306	4.319	0.071	
	Y × I	0.247	1	0.247	0.462	0.516	
	Error	4.271	8	0.534			
	Total	1739.005	12				
Branch number	Y	11.643	1	11.643	48.939	0.000	**
	I	6.337	1	6.337	26.635	0.001	**
	Y × I	0.032	1	0.032	0.135	0.723	
	Error	1.903	8	0.238			
	Total	807.883	12				
Plant height (cm)	Y	124.099	1	124.099	85.049	0.000	**
	I	222.655	1	222.655	152.592	0.000	**
	Y × I	8.961	1	8.961	6.142	0.038	*
	Error	11.673	8	1.459			
	Total	82,149.674	12				
Root fresh weight (g)	Y	46.217	1	46.217	62.788	0.000	**
	I	51.295	1	51.295	69.687	0.000	**
	Y × I	4.142	1	4.142	5.627	0.045	*
	Error	5.889	8	0.736			
	Total	1807.576	12				
Shoot fresh weight (g)	Y	926.642	1	926.642	327.580	0.000	**
	I	785.863	1	785.863	277.813	0.000	**
	Y × I	19.892	1	19.892	7.032	0.029	*
	Error	22.630	8	2.829			
	Total	119,456.143	12				
Root rot disease index	Y	0.307	1	0.307	433.694	0.000	**
	I	0.276	1	0.276	389.694	0.000	**
	Y × I	0.024	1	0.024	34.306	0.000	**
	Error	0.006	8	0.001			
	Total	9.080	12				

Y represents continuous cropping regimes. I represents inoculated with *R. intraradices*.

*Factors are significant at *P* < 0.05 level.

**Factors are significant at *P* < 0.01 level.

### Effects of *R. intraradices* and soybean continuous cropping on disease index of soybean root rot

The disease index of soybean root rot was significantly decreased by the inoculation of *R. intraradices* (Table 2). For instance, the disease index was 1.5 times for Non1Y in comparison with In1Y. Furthermore, the disease index of soybean root rot was significantly affected by soybean continuous cropping. There were significant differences in the disease index of soybean root rot under different continuous cropping regimes. As shown in Table 2, the disease index in Non1Y was the highest. However, the disease index of soybean root rot had nonsignificant differences between Non0Y and In1Y. It also showed that the interactive effects of the inoculation of *R. intraradices* and continuous cropping regimes were significantly affected the disease index of soybean root rot (*P* < 0.01) (Table 3).

### Composition of the rhizosphere bacterial communities

The diversity indices of bacteria in different samples were shown in Tables 4 and 5. Total 905,738 sequences with an average of 226,435 high-quality bacterial sequences per sample were obtained from the three replicates of the four rhizosphere soil samples which clustered into 1946 OTUs at a similarity level of 97%. There was no significant effect on OTU, Ace, Chao1 and Good’s coverages in the diversity indices of bacteria. Good’s coverages of the four libraries were greater than 0.999, indicating that the sequencing depth of all the soil samples was sufficient to represent the rhizosphere bacterial communities (Table 4). The variance analysis of the factorial design showed that the Simpson and Shannon indexes were significantly affected by the inoculation of *R. intraradices* and continuous cropping regimes (*P* < 0.01). It also showed that the interactive effects of them (*P* < 0.05) (Table 5). The Chao1 indexes of the four rhizosphere soil samples were 1900.86–1923.85. The Shannon indexes showed variations of 6.3742–6.5877 in the four rhizosphere soil samples. The relatively high Chao1 and Shannon indexes indicated that the bacterial diversity in the rhizosphere soil was high. Moreover, the Simpson index was the Highest in Non1YSB, while the opposite result occurred for the Ace index. The results showed that the bacterial diversity levels in the rhizosphere soil of continuous cropping soybean were lower than those in non-continuous cropping soybean. Furthermore, it showed that inoculation of *R. intraradices* could increase the bacterial diversity in rhizosphere soil of soybean.

**Table 4:** Factorial design results of diversity indices of bacteria and fungi in different samples.

	Treatment number	1	2	3	4
Bacteria	Continuous cropping regimes	0Y	0Y	1Y	1Y
	AM fungal treatments	Non	In	Non	In
	OTU	1923 ± 5^a^	1919 ± 10^a^	1925 ± 8^a^	1922 ± 3^a^
	Ace	1901.96 ± 8.27^a^	1905.36 ± 6.05^a^	1894.94 ± 10.75^a^	1896.64 ± 4.30^a^
	Chao1	1911.32 ± 11.65^a^	1923.85 ± 6.23^a^	1900.86 ± 2.73^a^	1911.03 ± 11.38^a^
	Simpson	0.0037 ± 0.0001^c^	0.0035 ± 0.0001^c^	0.0059 ± 0.0001^a^	0.0048 ± 0.0002^b^
	Shannon	6.5776 ± 0.0183^a^	6.5877 ± 0.0065^a^	6.3742 ± 0.0109^c^	6.4504 ± 0.0211^b^
	Coverage	0.9999 ± 0.0001^a^	0.9998 ± 0.0002^a^	0.9999 ± 0.0001^a^	0.9999 ± 0.0001^a^
Fungi	OTU	343 ± 57^b^	567 ± 34^a^	227 ± 15^c^	226 ± 17^c^
	Ace	265.64 ± 62.59^ab^	330.03 ± 32.23^a^	203.84 ± 24.80^b^	197.29 ± 15.32^b^
	Chao1	243.78 ± 46.31^ab^	312.54 ± 33.21^a^	196.59 ± 30.48^b^	196.92 ± 21.84^b^
	Simpson	0.1518 ± 0.0587^a^	0.0199 ± 0.0086^b^	0.1823 ± 0.0455^a^	0.1702 ± 0.0285^a^
	Shannon	2.7457 ± 0.8105^b^	4.6970 ± 0.1086^a^	2.5153 ± 0.1474^b^	2.5170 ± 0.4481^b^
	Coverage	0.9990 ± 0.0001^a^	0.9993 ± 0.0002^a^	0.9991 ± 0.0001^a^	0.9991 ± 0.0001^a^

0Y and 1Y represent 0 year and 1 year of continuous cropping, respectively.

Non represents non-inoculated with *R. intraradices*. In represents inoculated with *R. intraradices*.

Different letters indicate significant differences from different treatments (*P* < 0.05).

**Table 5:** Variance analysis of the factorial design of diversity indices of bacteria and fungi in different samples.

	Index	Source	Sum of squares	df	Mean square	F-value	*P*-value	Significance level
Bacteria	OTU	Y	14.083	1	14.083	0.302	0.597	
		I	44.083	1	44.083	0.946	0.359	
		Y × I	2.083	1	2.083	0.045	0.838	
		Error	372.667	8	46.583			
		Total	44,348,663.000	12				
	Ace	Y	185.811	1	185.811	3.109	0.116	
		I	19.507	1	19.507	0.326	0.583	
		Y × I	2.168	1	2.168	0.036	0.854	
		Error	478.096	8	59.762			
		Total	43,308,146.489	12				
	Chao1	Y	406.469	1	406.469	5.220	0.052	
		I	386.467	1	386.467	4.963	0.056	
		Y × I	4.177	1	4.177	0.054	0.823	
		Error	622.985	8	77.873			
		Total	43,859,565.082	12				
	Simpson	Y	9.187E−6	1	9.187E−6	525.000	0.000	**
		I	1.268E−6	1	1.268E−6	72.429	0.000	**
		Y × I	6.075E−7	1	6.075E−7	34.714	0.000	**
		Error	1.400E−7	8	1.750E−8			
		Total	0.000	12				
	Shannon	Y	0.087	1	0.087	370.000	0.000	**
		I	0.006	1	0.006	23.740	0.001	**
		Y × I	0.003	1	0.003	13.927	0.006	**
		Error	0.002	8	0.000			
		Total	506.704	12				
	Coverage	Y	7.500E−9	1	7.500E−9	0.900	0.371	
		I	7.500E−9	1	7.500E−9	0.900	0.371	
		Y × I	7.500E−9	1	7.500E−9	0.900	0.371	
		Error	6.667E−8	8	8.333E−9			
		Total	11.996	12				
Fungi	OTU	Y	156,636.750	1	156,636.750	127.373	0.000	**
		I	37,296.750	1	37,296.750	30.329	0.001	**
		Y × I	37,968.750	1	37,968.750	30.875	0.001	**
		Error	9838.000	8	1229.750			
		Total	1,635,067.000	12				
	Ace	Y	28,384.359	1	28,384.359	19.555	0.002	**
		I	2509.099	1	2509.099	1.729	0.225	
		Y × I	3774.363	1	3774.363	2.600	0.146	
		Error	11,612.047	8	1451.506			
		Total	791,487.547	12				
	Chao1	Y	19,880.322	1	19,880.322	17.088	0.003	**
		I	3580.071	1	3580.071	3.077	0.117	
		Y × I	3511.999	1	3511.999	3.019	0.121	
		Error	9307.072	8	1163.384			
		Total	712,912.236	12				
	Simpson	Y	0.025	1	0.025	15.318	0.004	**
		I	0.016	1	0.016	9.717	0.014	*
		Y × I	0.011	1	0.011	6.725	0.032	*
		Error	0.013	8	0.002			
		Total	0.270	12				
	Shannon	Y	4.358	1	4.358	19.557	0.002	**
		I	2.861	1	2.861	12.839	0.007	**
		Y × I	2.851	1	2.851	12.795	0.007	**
		Error	1.782	8	0.223			
		Total	128.571	12				
	Coverage	Y	7.500E−9	1	7.500E−9	0.429	0.531	
		I	6.750E−8	1	6.750E−8	3.857	0.085	
		Y × I	6.750E−8	1	6.750E−8	3.857	0.085	
		Error	1.400E−7	8	1.750E−8			
		Total	11.979	12				

Y represents continuous cropping regimes. I represents inoculated with *R. intraradices*.

*Factors are significant at *P* < 0.05 level.

**Factors are significant at *P* < 0.01 level.

**Figure 1:**
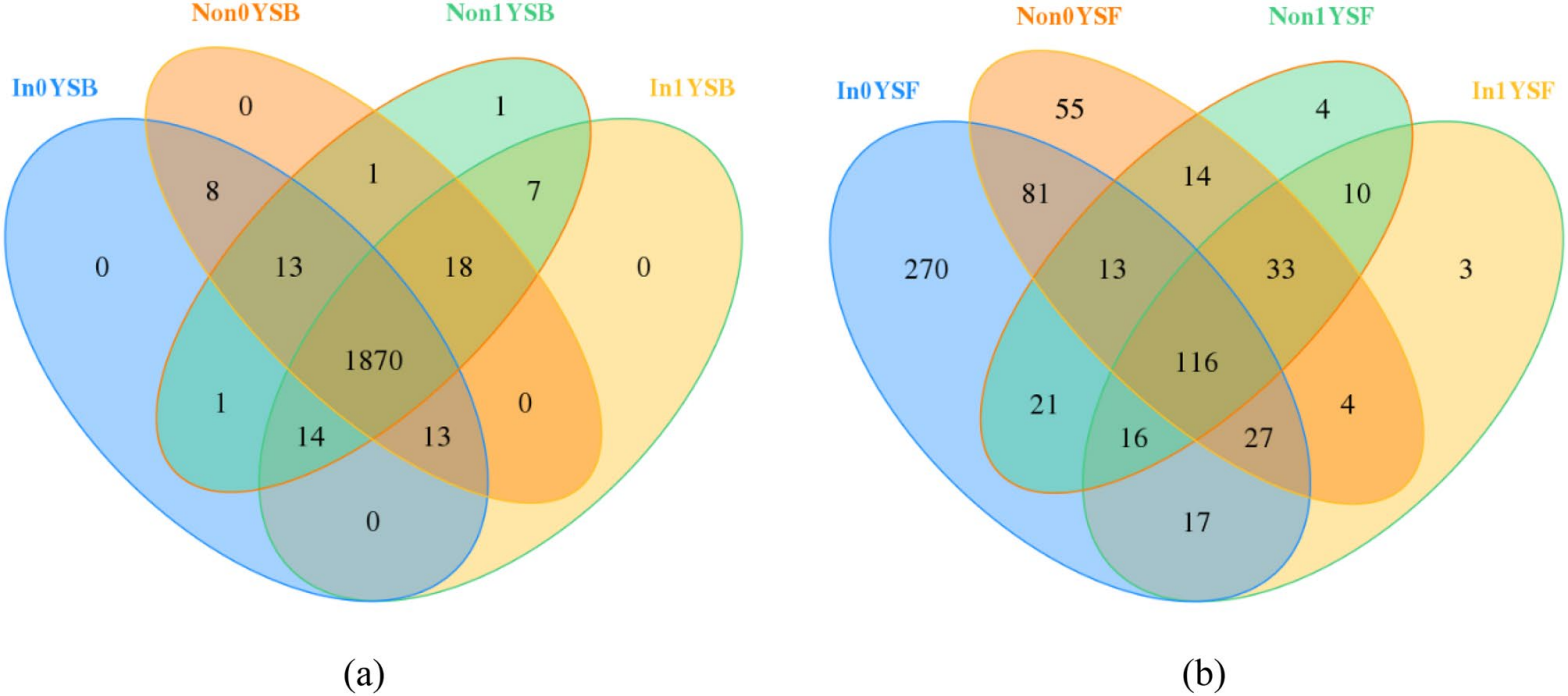
The VENN diagrams of the four samples according to bacterial (**a**) and fungal (**b**) diversity. Non represents non-inoculated with *R. intraradices*. In represents inoculated with *R. intraradices*. 0Y and 1Y represent 0 year and 1 year of continuous cropping, respectively. SB represents bacteria in rhizosphere soil. SF represents fungi in rhizosphere soil.

The distribution of OTUs was evaluated using VENN diagrams (Fig. 1a). As shown in Fig. 1a, there were differences in the amount of shared OTUs among rhizosphere soil samples inoculated or non-inoculated with *R. intraradices*. Consistent with the alpha diversity, there were more shared OTUs in the rhizosphere soil samples.

**Figure 2:**
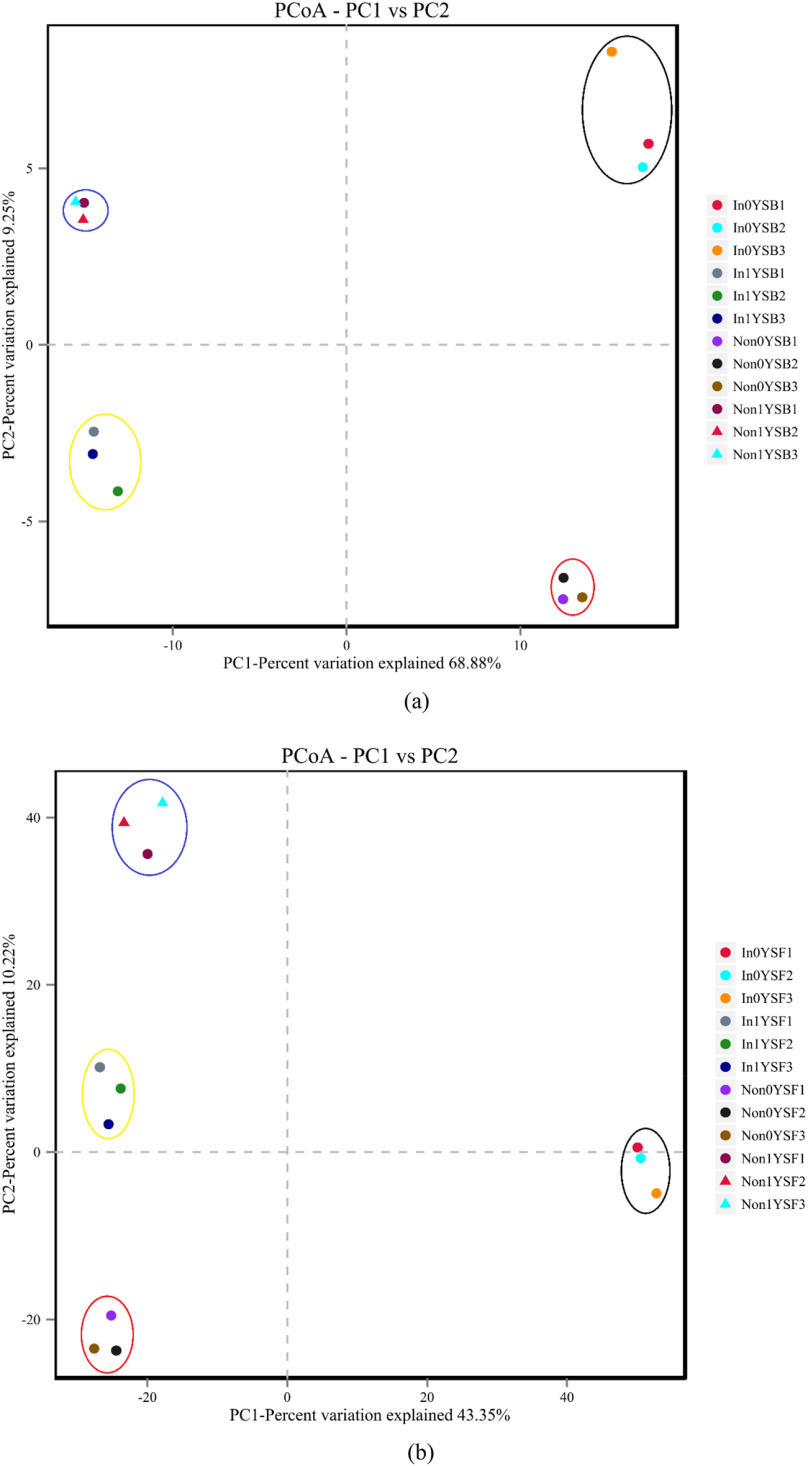
Principal coordinates analysis (PCoA) of the composition of bacterial (**a**) and fungal (**b**) communities. Non represents non-inoculated with *R. intraradices*. In represents inoculated with *R. intraradices*. 0Y and 1Y represent 0 year and 1 year of continuous cropping, respectively. SB represents bacteria in rhizosphere soil. SF represents fungi in rhizosphere soil. The numbers 1, 2 and 3 represent three repetitions.

The difference or similarity of the composition of bacterial communities across all rhizosphere soil samples was illustrated using PCoA analysis on the basis of Bray–Curtis distance (Fig. 2a). The plot clearly showed that all rhizosphere soil samples were separated into four groups. It indicated that the composition of bacterial communities in both non-inoculated and inoculated with *R. intraradices* soils was distinctly different between the two continuous cropping regimes.

**Figure 3:**
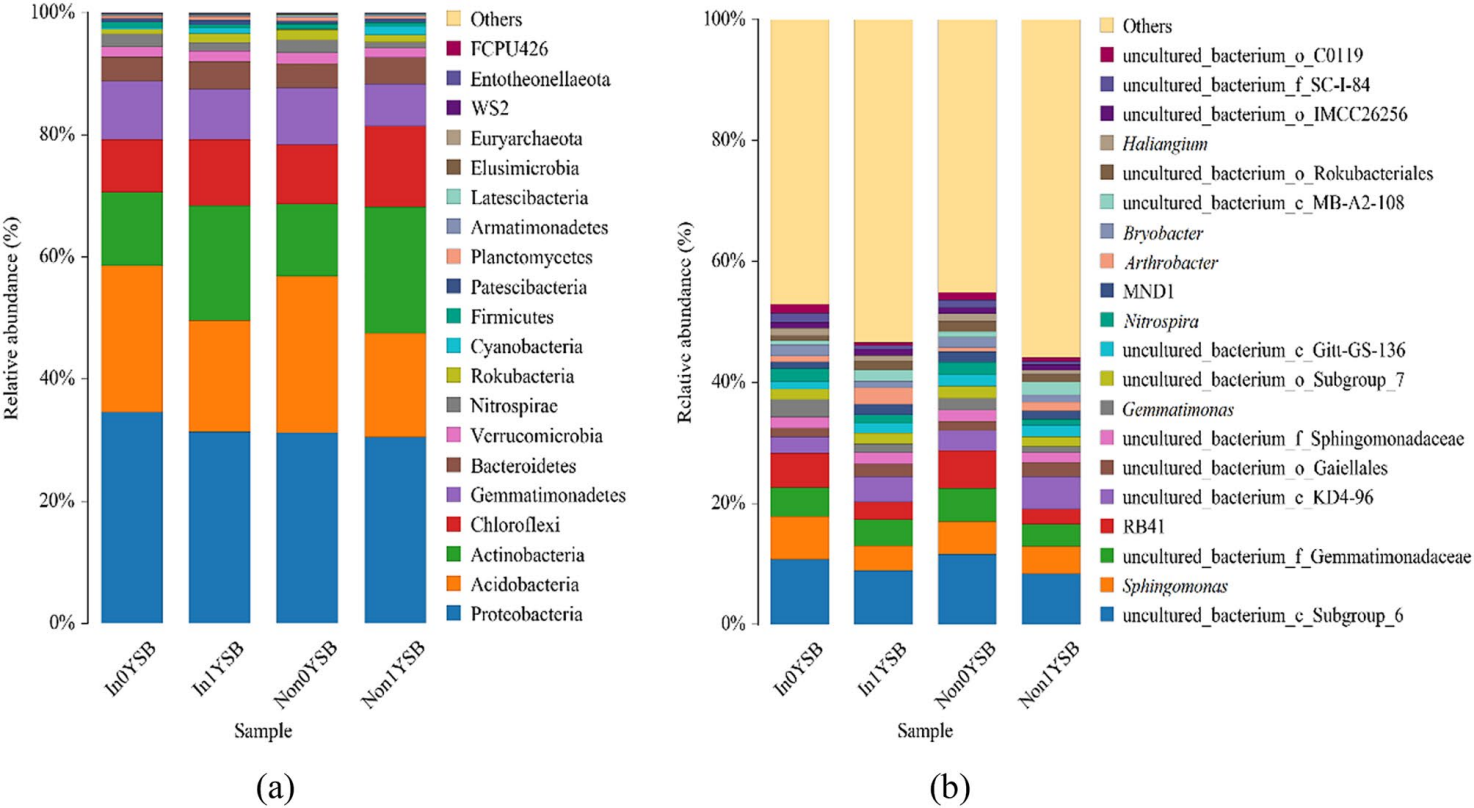
The composition of bacterial communities at the phylum (**a**) and genus (**b**) levels. Non represents non-inoculated with *R. intraradices*. In represents inoculated with *R. intraradices*. 0Y and 1Y represent 0 year and 1 year of continuous cropping, respectively. SB represents bacteria in rhizosphere soil.

The three replicates of each rhizosphere soil sample were mixed into one sample. From the four rhizosphere soil samples, twenty different bacterial phyla were detected (Fig. 3a). As shown in Fig. 3a, the most dominant bacterial phyla in the four rhizosphere soil samples were Proteobacteria, Acidobacteria and Actinobacteria. Chloroflexi, Gemmatimonadetes and Bacteroidetes were the following dominant phyla in the rhizosphere soil samples except for In0YSB. Gemmatimonadetes (9.53%) was the fourth dominant phylum, followed by Chloroflexi (8.65%) and Bacteroidetes (3.99%) in In0YSB. The relative abundances of the other dominant phyla in the four rhizosphere soil samples varied under the effects of *R. intraradices* and continuous cropping. Notably, the relative abundance of Verrucomicrobia had an advantage in the rhizosphere soil samples except Non1YSB. In addition, the relative abundance of Nitrospirae increased from 0.97% in Non1YSB to 2.12% in In0YSB.

At the genus level, the most dominant genus was uncultured_bacterium_c_Subgroup_6 in all the rhizosphere soil samples (Fig. 3b). The relative abundance of *Sphingomonas* (7.12%) in In0YSB was much higher than the other three rhizosphere soil samples. Furthermore, *Gemmatimonas* (2.90%), *Nitrospira* (2.12%), *Bryobacter* (1.86%), *Haliangium* (1.23%), and *Arthrobacter* (0.95%) were also detected in In0YSB. Nevertheless, the relative abundance of *Gemmatimonas* decreased and remained at about 1.01% in Non1YSB. Due to the inoculation of *R. intraradices*, the relative abundances of *Gemmatimonas*, *Nitrospira* and *Arthrobacter* in In0YSB and In1YSB were higher than that in Non0YSB and Non1YSB, respectively. It showed that *R. intraradices* might have effects on the composition of bacterial communities in the rhizosphere soil samples in this study. As shown in Fig. 3b there were also differences in the relative abundances of these dominant genera between the rhizosphere soil samples of continuous cropping and non-continuous cropping soybean. The results showed that the composition of bacterial communities was affected by *R. intraradices* and soybean continuous cropping.

**Figure 4:**
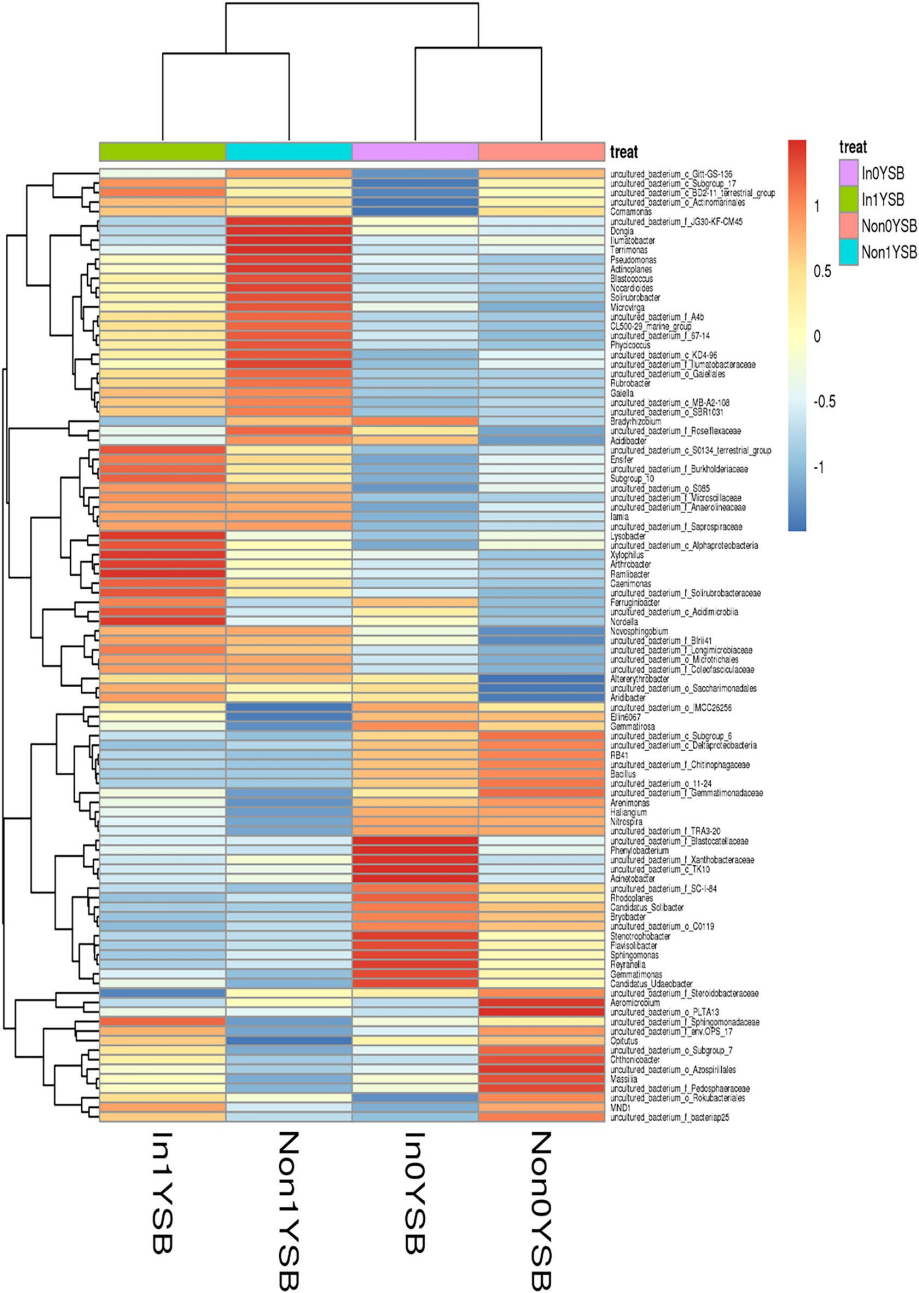
Heat map of the 100 most abundant bacterial genera. Non represents non-inoculated with *R. intraradices*. In represents inoculated with *R. intraradices*. 0Y and 1Y represent 0 year and 1 year of continuous cropping, respectively. SB represents bacteria in rhizosphere soil.

According to the heatmap diagram of the bacterial communities at the genus level, the four rhizosphere soil samples were divided into two clusters: Non0YSB and In0YSB clustered together; Non1YSB and In1YSB clustered together, indicating that the bacterial communities between the two rhizosphere soil samples were similar (Fig. 4). The results also demonstrated that both *R. intraradices* and continuous cropping could affect the dominant genera and their relative abundances in the four rhizosphere soil samples.

### Composition of the rhizosphere fungal communities

The diversity indices of fungi in different samples were shown in Tables 4 and 5. The variance analysis of the factorial design of the diversity indices of fungi showed that OTU, Simpson and Shannon indexes were significantly affected by the inoculation of *R. intraradices* and continuous cropping regimes (*P* < 0.01). It also showed that the interactive effects of them (*P* < 0.05) (Table 5). A total number of 860,192 sequences with an average of 215,048 high-quality fungal sequences per sample were obtained from the three replicates of the four rhizosphere soil samples. The fungal diversity in the rhizosphere soil of continuous cropping soybean showed a similar trend as that of bacterial diversity (Table 4). There was no significant effect on the Good’s coverages in the diversity indices of fungi. The fungal sequences clustered into 684 OTUs at a similarity level of 97%. As shown in Table 4, the Good’s coverages of the four libraries were also greater than 0.999. The Ace and Chao1 indexes were significantly affected by continuous cropping regimes (*P* < 0.05) (Table 5). The Chao1 and Shannon indexes showed variations of 196.59–312.54 and 2.5153–4.6970 in the four rhizosphere soil samples, respectively. In addition, the Simpson index was the lowest in In0YSF, and the Ace index was the highest in In0YSF. It suggested that the fungal diversity levels in the rhizosphere soil of continuous cropping soybean were lower than those in non-continuous cropping soybean, and the same result occurred for the bacterial diversity levels. In addition, the results showed that inoculation of *R. intraradices* could increase the fungal diversity in rhizosphere soil of soybean.

As shown in Fig. 1b, the rhizosphere soil samples inoculated or non-inoculated with *R. intraradices* were also different in the amount of shared OTUs. However, there was fewer shared OTUs in the rhizosphere soil samples compared with that of bacteria.

PCoA was used to analyze the differences in the composition of fungal communities between rhizosphere soil samples (Fig. 2b). Three replicates clustered closely, indicating the reproducibility of the fungal community profiles. The plot clearly showed that all rhizosphere soil samples were separated into four groups. It indicated that the composition of fungal communities changed greatly by the effects of inoculation with *R. intraradices* and continuous cropping regimes.

**Figure 5:**
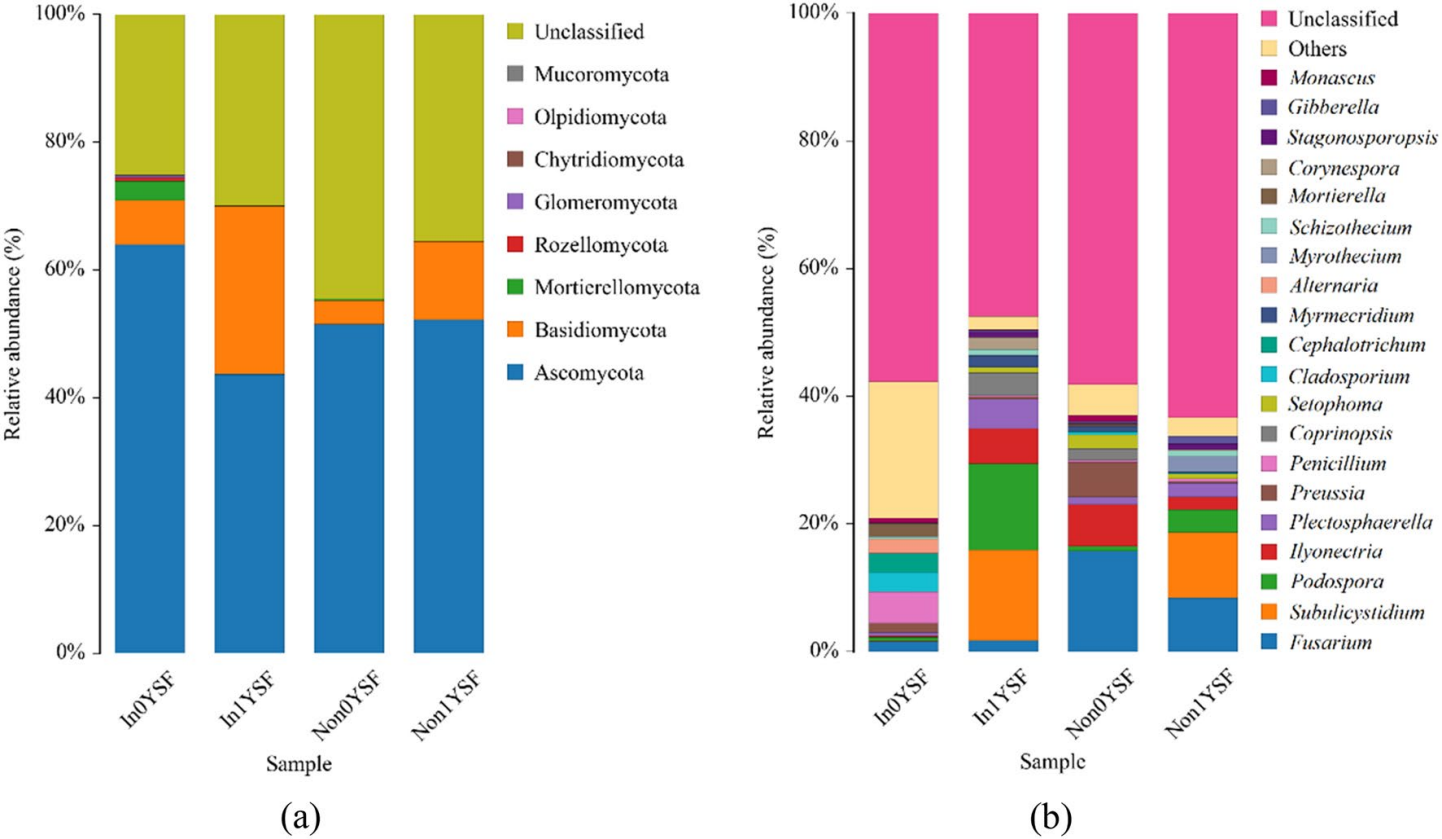
The composition of fungal communities at the phylum (**a**) and genus (**b**) levels. Non represents non-inoculated with *R. intraradices*. In represents inoculated with *R. intraradices*. 0Y and 1Y represent 0 year and 1 year of continuous cropping, respectively. SF represents fungi in rhizosphere soil.

Fungal communities along with different rhizosphere soil samples were analyzed to study the variation among different treatments. A total of eight phyla were identified in the four rhizosphere soil samples, including Ascomycota, Basidiomycota, Mortierellomycota, Rozellomycota, Glomeromycota, Chytridiomycota, Olpidiomycota, and Mucoromycota (Fig. 5a). As shown in Fig. 5a, the relative abundances of the eight dominant phyla varied under the effects of *R. intraradices* and soybean continuous cropping. Ascomycota (accounted for more than 43.66% of the total amount) was the most dominant phylum in the four rhizosphere soil samples. Basidiomycota and Mortierellomycota were the second and third dominant phyla in the four rhizosphere soil samples, respectively. However, the relative abundances of Ascomycota and Mortierellomycota in In0YSF were higher than that in the other rhizosphere soil samples. In addition, the relative abundances of Rozellomycota, Glomeromycota, Chytridiomycota, Olpidiomycota, and Mucoromycota were very low.

At the genus level, there were differences in the relative abundances of the dominant genera among the four rhizosphere soil samples. As shown in Fig. 5b, the most dominant genus was *Subulicystidium* in In1YSF and Non1YSF. However, *Fusarium* was the most dominant genus in In0YSF and Non0YSF. Interestingly, the relative abundance of *Fusarium* decreased from 15.72% in Non0YSF to 1.58% in In0YSF. The relative abundance of *Fusarium* in Non1YSF and In1YSF showed a similar trend*.* Some plant pathogenic fungi, such as *Ilyonectria*, *Plectosphaerella*, *Cladosporium*, and *Corynespora*, were also detected in the four rhizosphere soil samples. However, there were differences in their relative abundances. Furthermore, the second dominant genus was *Podospora* in In1YSF and Non1YSF, which accounted for at least 3.47% of the total fungal abundance. *Ilyonectria* (6.48%) and *Penicillium* (4.90%) were the second dominant genera in Non0YSF and In0YSF, respectively. The results showed that *R. intraradices* and soybean continuous cropping might have effects on the composition of fungal communities, which were similar to those observed at the phylum level.

**Figure 6:**
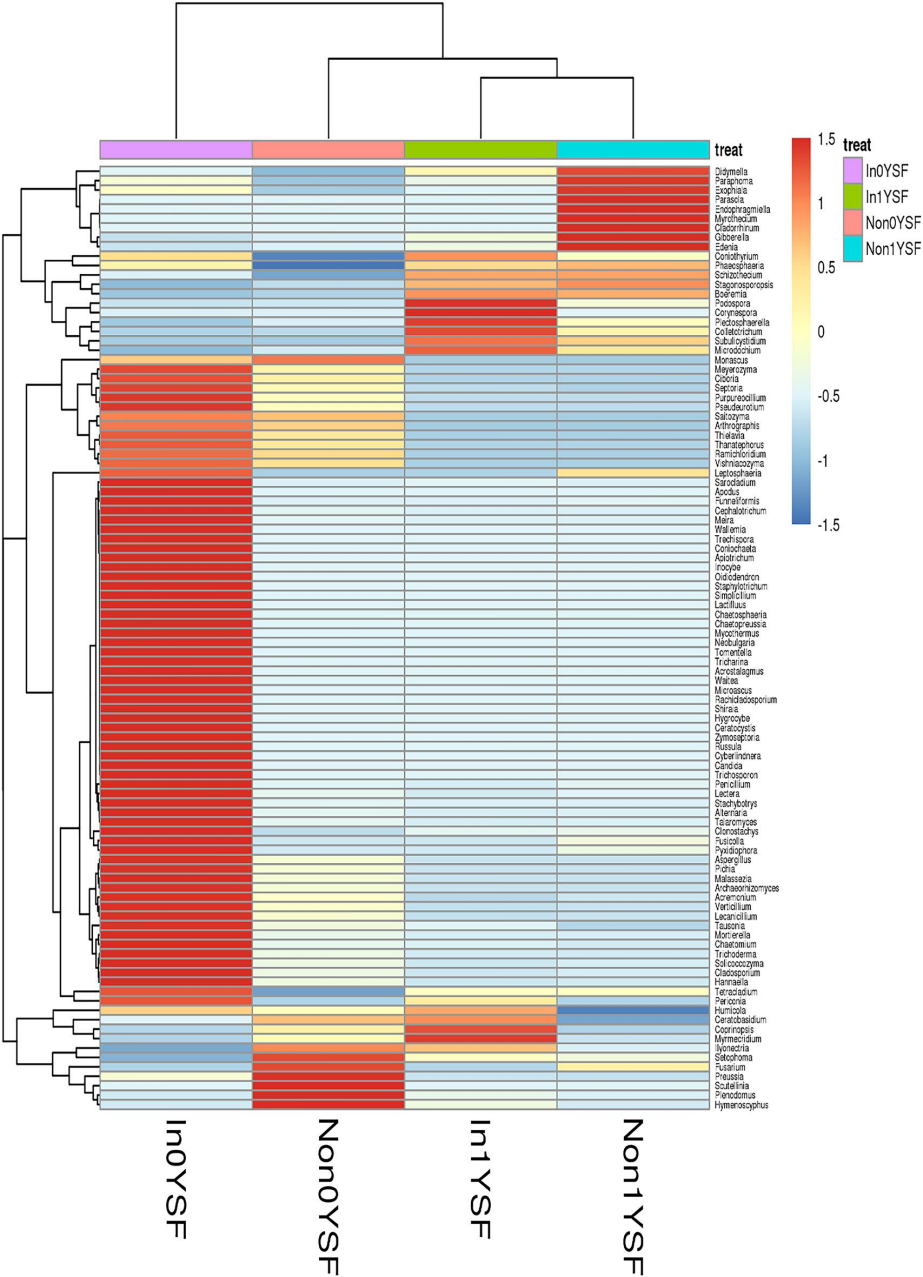
Heat map of the 100 most abundant fungal genera. Non represents non-inoculated with *R. intraradices*. In represents inoculated with *R. intraradices*. 0Y and 1Y represent 0 year and 1 year of continuous cropping, respectively. SF represents fungi in rhizosphere soil.

The top hundred OTUs divided the four rhizosphere soil samples into the following two groups: In0YSF did not cluster with other rhizosphere soil samples; Non0YSF, Non1YSF and In1YSF clustered together, indicating their similar core function in shaping the composition of fungal communities (Fig. 6). It was consistent with the composition of fungal communities at the genus level. In addition, it also demonstrated that both *R. intraradices* and soybean continuous cropping could affect the dominant genera and their relative abundances in the four rhizosphere soil samples.

## Discussion

The purpose of this study was to investigate the effects of *R. intraradices* on soybean growth/yield, root rot disease index and the composition of microbial communities in the rhizosphere soil of continuous cropping soybean at reproductive R8 stage. It has been reported that AM fungi can increase plant biomass and resistance to pathogenic microorganisms, and reduce the severity of diseases^36^. The use of AM fungi provides a sustainable choice for crop disease control. Colonization of AM fungi in plant roots reduces many diseases and improves plant resistance to pathogenic microorganisms^37^. This is because AM fungi compete with pathogenic microorganisms for nutrients and space, and protect root tissue from pathogenic microorganisms^38^.

In this study, it showed that inoculation of *R. intraradices* significantly increased the 100-seed weight, seed-yield per plant, yield per 0.04 hectare, pods per plant, seed number per plant, branch number, plant height, and fresh weight of root and shoot compared with the corresponding non-inoculated soybean plants. The disease index of soybean root rot was significantly decreased by the inoculation of *R. intraradices* (Table 2). AM fungi can increase root biomass, which can compensate for the damage caused by pathogens^39^. In addition, AM fungi can compete with soilborne pathogenic fungi for the colonization sites in the plant roots, because they occupy similar root tissues^40^. The establishment of mycorrhizal symbiosis by pre-activating the plant defense response can make plants more responsive to pathogen attacks^36^. When AM fungi colonize plant roots, morphological changes occur inside the host plant, such as the increase of lignification of the cell wall, which may contribute to biological conservation^41^.

The composition of rhizosphere microbial communities of AM fungi infected plant roots is different from that of non-mycorrhizal roots^42^. A large number of studies showed that there were bacterial communities closely related to AM fungal spores, hyphae and mycorrhizal roots in the mycorrhizosphere. Mycorrhiza helper bacteria and plant growth promoting rhizobacteria (PGPR) play an important role in promoting AM fungi activity^43^. Bacteria in the mycorrhizosphere can promote AM fungal spore germination, mycelial growth and mycorrhizal colonization^44^. The spatially confined structure of the mycorrhizosphere allows rhizobacteria to achieve abnormally high cell densities^45^. In addition, several genera of the rhizobacteria can promote plant growth^46,47^. Like pathogenic microorganisms, beneficial microorganisms also need to evade plant immune responses, to establish a long-term, close and mutually beneficial interaction with the host^48,49^. As shown in Fig. 3a, the most dominant bacterial phyla in the four rhizosphere soil samples were Proteobacteria, Acidobacteria and Actinobacteria. However, there were differences in the relative abundances of these dominant genera among the four rhizosphere soil samples.

Soil type is also an important factor that determines the composition of rhizosphere microbial communities^50^. In this study, the results showed that the composition of bacterial and fungal communities in soybean continuous cropping soil was different from that in non-continuous cropping soil. As shown in Fig. 5a, Ascomycota, Basidiomycota and Mortierellomycota were the most dominant fungal phyla in the four rhizosphere soil samples. The most dominant genus was *Subulicystidium* in In1YSF and Non1YSF. However, *Fusarium* was the most dominant genus in In0YSF and Non0YSF. Plant root diseases can be controlled by controlling native microorganisms to reduce the abundance of pathogenic microorganisms^51^. The highly specific microbial communities in the plant rhizosphere may have important effects on plant pathogenic microorganisms. By favoring specific microbial populations and reducing the abundance of other microbial populations by inoculation of AM fungi, the abundance of plant pathogenic microorganisms may be controlled^52^. The relative abundance of *Fusarium* decreased from 15.72% in Non0YSF to 1.58% in In0YSF by the inoculation of *R. intraradices* (Fig. 5b).

Arbuscular mycorrhizal fungi and their symbiosis with hosts can reduce damage caused by pathogenic microorganisms^52^. Arbuscular mycorrhizal fungi and soil-borne pathogenic fungi occupy similar root tissues. If they colonize at the same time, they will directly compete for root space^53^. The lignification of cell walls increased after AM fungi infected plant roots, which may contribute to biological protection^54^. The results showed that inoculation of *R. intraradices* could increase soybean yield, decrease root rot disease index and change the composition of microbial communities in the rhizosphere soil of continuous cropping soybean at the R8 stage. In addition, the results of this study would contribute to isolating and identifying the beneficial microorganisms in the rhizosphere soil for alleviating the obstacles of soybean continuous cropping.

## Conclusions

This work first demonstrated that *R. intraradices* can directly alter the soybean growth/yield, root rot disease index and the composition of microbial communities in the rhizosphere soil of continuous cropping soybean at the R8 stage. The 100-seed weight, seed-yield per plant, yield per 0.04 hectare, pods per plant, seed number per plant, branch number, plant height, and fresh weight of root and shoot were significantly increased by the inoculation of *R. intraradices*. Inoculation of *R. intraradices* and soybean continuous cropping significantly decreased and increased the disease index of soybean root rot, respectively. Furthermore, inoculation of *R. intraradices* could increase the microbial diversity in rhizosphere soil of soybean. The relative abundances of several microbial phyla varied under the effects of *R. intraradices* and continuous cropping regimes. Proteobacteria and Ascomycota were the most dominant bacterial and fungal phylum in all samples, respectively. The purpose of this study was to evaluate the biocontrol potential of *R. intraradices* against soybean root rot disease as well as its role in alleviating the obstacles of soybean continuous cropping.

## Data Availability

The datasets used and/or analysed during the current study available from the corresponding author on reasonable request.
